# A Multicentric Audit to Reevaluate the Guidelines Adherence in Computed Tomography of Kidneys, Ureters, and Bladder (CT-KUB) X-ray Imaging in Jordan

**DOI:** 10.7759/cureus.53634

**Published:** 2024-02-05

**Authors:** Sallam Alrosan, Mohammad Abu-Jeyyab, Marah Alabbasi, Huda Baidoun, Abdel Rahman Bani Yassin, Shahd Mansour, Zaid Al-Rabadi, Basil Aldiabat, Yala Jawazneh, Salwa Azzawi, Malak Alkatib, Mohammad Al Mse'adeen

**Affiliations:** 1 Internal Medicine, Saint Luke’s Health System, Kansas City, USA; 2 Medical School, Mutah University, Al-Karak, JOR; 3 Medical School, Unversity of Jordan, Amman, JOR; 4 Medical School, Jordan University of Science and Technology, Irbid, JOR; 5 Medical School, University of Jordan, Amman, JOR

**Keywords:** computrized topography, imaging modalities, over-scanning, renal ct scan, audit

## Abstract

Background

With the increasing use of imaging techniques involving ionizing radiation, the area of the body scanned should be restricted to what is required to answer the clinical question. Therefore, this is a retrospective audit that intends to evaluate the presence of overscanning in renal computed tomography (CT) scan images during the process of evaluation for urinary symptoms.

Objective

This study aims to reduce the unnecessary scan length and exposure to radiation in patients who undergo CT scans for urinary symptoms.

Materials and Methods

In two months duration, patients from different clinics underwent CT imaging, and the resulting radiographic images were collected and analyzed. Overscanning was defined to be more than 10% of the total scan. Subsequently, the total length of the CT scan was measured which is used to measure the unnecessary overscan above the highest kidney margin as a percentage of the total length.

Results

Out of the 88 patients who were evaluated, 100% did not meet the guidelines for renal CT imaging and were exposed to a high radiation dose. However, the minimum percentage of overscanned patients was 20-40%.

Conclusion

A significant number of scans demonstrated surplus overscanning above the highest kidney. Therefore, recognizing the suitable anatomical landmarks for scanning and establishing a follow-up audit are suggested measures to minimize the noxious effects of radiation exposure.

## Introduction

Despite the clinical advantages of CT imaging, as well as its valuable clinical applications, persistent concerns are present regarding the potential risks linked to ionizing radiation exposure. In order to evaluate the potential risk of harmful biological effects caused by exposure to ionizing radiation, it is important to comprehend the mechanisms involved in radiation damage and repair [1]. Individuals experiencing renal colic face a 7% chance of annual recurrence [2], necessitating multiple CT scans throughout their lifetime. CT imaging-related radiation exposure is associated with the age at which renal symptoms appear, with the highest exposure estimated in the youngest patients [3]. Recent research suggests ultrasound as an effective alternative for diagnostics, particularly in pediatric and pregnant ladies [4]. A formal renal ultrasound, involving a thorough examination of the renal and urologic systems by a radiologist or radiology technician, presents several advantages over other alternative diagnostic imaging modalities. In assessing patients with acute urologic disorders, the emergency physician (EP) has multiple options for imaging modalities, including renal ultrasound. Renal ultrasound provides excellent anatomical detail without exposing the patient to the risk of radiation or contrast agents. While it has limitations in assessing renal function, it serves as a crucial alternative to helical CT scanning, especially in the evaluation of renal colic [5]. Considering both cost and radiation factors, our recommendation is to prioritize ultrasound (US) as the initial diagnostic modality for detecting ureteral stones. However, utilization of CT scans is suggested in cases where ultrasound is unavailable or yields non-diagnostic results [6]. CT overscanning has the potential to provide a concern due to higher ionizing radiation exposure. Patients are subjected to higher levels of ionizing radiation during a CT scan than they would be during a standard X-ray. While there is no evidence of long-term harm from the modest radiation doses used in CT scans, there may be a slight increase in the risk of cancer with considerably higher radiation doses. Therefore, in order to protect patients and reduce needless radiation exposure, it is essential to take these considerations into account when doing CT scans [7]. Clinical auditing, as a quality improvement process, aims to enhance patient care and outcomes through systematically reviewing care against specific criteria and implementing necessary changes. The structure, processes, and outcomes of care are carefully chosen and systematically evaluated against explicit criteria. If needed, changes are implemented at the individual team or service level, and ongoing monitoring is implemented to confirm improvements in healthcare delivery [8]. This study aims to reduce the unnecessary scan length and exposure to radiation in patients who undergo CT scans in the process of evaluation for urinary symptoms in 3 teaching hospitals and referral centers in Jordan.

## Materials and methods

Over the course of two months, this clinical audit was carried out in three teaching hospitals and referral centers in Jordan. It is a retrospective clinical audit. Two of these hospitals, A and C, are situated in the city of Amman, the capital of Jordan, and the third hospital B is situated in the southern region of the country. In order to determine the extent of the CT scan overscanning that was performed for the purpose of investigating urinary complaints, the audit was conducted. In Jordan, the standard computed tomography of kidneys, ureters, and bladder (CT-KUB) is low-dose CT (LDCT) since LDCT has a lower risk of radiation-induced harm. The research project was given the go-ahead by the Ethics Committee of the School of Medicine at the University of Jordan, which assigned it the number JUH39-2023. The strategy, design, and consequences of this research were developed through the process of comprehensively reviewing the related literature.

Revaluation methods

After 5 months of educating and meetings with the staff in the radiology department, and hanging posters in different departments of the hospital including the radiology department, emergency department, and different hallways in the hospital (see Appendices) additional data was acquired from the 3 hospitals.

Data analysis

Data was analyzed using Statistical Package for Social Sciences (SPSS), version 26.0 (IBM Corp., Armonk, NY). Descriptive statistics were used to summarize frequencies, means, and percentages. A comparison between hospitals’ results was done using the chi-square test.

## Results

This audit was conducted in 3 teaching hospitals and referral centers in Jordan. It included 88 participants, of which 62.5% were males and 37.5% were females (Table 1) with a mean age of 45.5 years old and a standard deviation of (13.5) with a minimum age of 19 and a maximum age of 85. The 88 participants in this study were divided as follows: 10 patients (11.4%) were from hospital A, 14 patients (15.9%) were from hospital C, and 64 patients (72.7%) were from hospital B (Table 2).

**Table 1:** Gender frequencies among the patients Gender frequencies and percentages in the sample; N (%)

Valid	Frequency	Percent (%)	Valid percent	Cumulative percent
F	33	37.5%	37.5	37.5
M	55	62.5%	62.5	100.0
Total	88	100.0	100.0	-

**Table 2:** Hospital frequencies Number and percentages of patients in each hospital; N (%)

Valid	Frequency	Percent (%)	Valid percent	Cumulative percent
Hospital A	10	11.4%	11.4	11.4
Hospital C	14	15.9%	15.9	27.3
Hospital B	64	72.7%	72.7	100.0
Total	88	100.0	100.0	-

**Figure 1:**
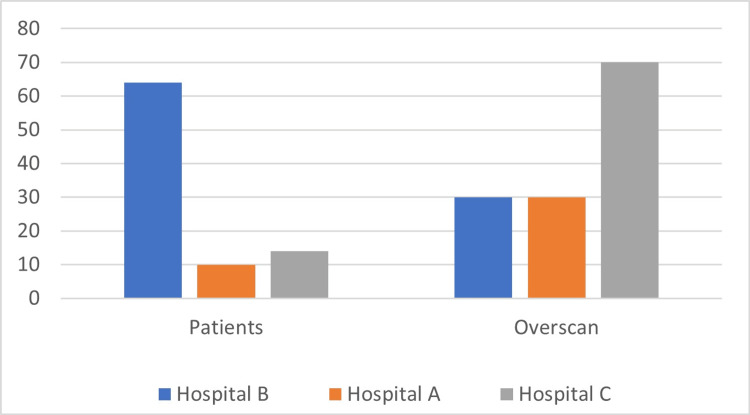
This figure shows patients’ distribution according to age and overscan percentages.

Figure 1 shows the distribution of the overscan percentage for each hospital before and after intervention.

The data was also organized according to age. It showed patients between the age of 41-60 with a percentage of 55.7% and patients between the age of 21-40 with a percentage of 30% (Table 3).

**Table 3:** Age frequencies among the patients Age frequencies and percentages in the sample; N (%) Intervals: 1=(0-20) years old, 2=(21-40) years old, 3=(41-60) years old, 4=(61-80) years old, 5=(81-100) years old

Valid	Frequency	Percent (%)	Valid percent	Cumulative percent
1	2	2.3%	2.3	2.3
2	27	30.7%	30.7	33.0
3	49	55.7%	55.7	88.6
4	8	9.1%	9.1	97.7
5	2	2.3%	2.3	100.0
Total	88	100.0	100.0	-

The data showed that 56 out of the 88 images (a percentage of 63%) were (20-40%) overscanned and only one image was more than 80% overscanned (Table 4).

**Table 4:** Overscanning frequencies in numbers Overscan frequencies and percentages in the sample; N (%)

Valid	Frequency	Percent (%)	Valid percent	Cumulative percent
1	1	1.1%	1.1	1.1
2	56	63.6%	63.6	64.8
3	17	19.3%	19.3	84.1
4	13	14.8%	14.8	98.9
5	1	1.1%	1.1	100.0
Total	88	100.0	100.0	-

There was a significant difference between hospitals in overscan percentages (p-value=0.00, considered significant p value<0.05). Hospital C has the highest records of CT-overscan as 85% of images were in the fourth interval which equals (61-80%) overscan. Seventy-five percent of the hospital A and hospital B images were in the second interval which is 21-40% overscan percentage (Table 5).

**Table 5:** Hospital overscan cross-tables Chi-square for overscan distribution in each hospital is represented as N (%) per hospital Key to overscan percentages on 5 intervals: 1=(0-20%), 2=(21-40%), 3=(41-60%), 4=(61-80%), 5=(81-100%)

		1	2	3	4	5	Total
Hospital	Hospital B	1 (1.5%)	48 (75%)	14 (22%)	1 (1.5%)	0 (0%)	64 (100%)
	Hospital C	0 (0%)	0 (0%)	1 (7.14%)	12 (85.72%)	1 (7.14%)	14 (100%)
	Hospital A	0 (0%)	8 (80%)	2 (20%)	0 (0%)	0 (0%)	10 (100%)
Total	-	1 (1.135%)	56 (63.64%)	17 (19.32%)	13 (14.77%)	1 (1.135%)	88 (100%)

Reaudit results

The data showed major improvement in which fewer patients were overscanned. Hospital C showed the best improvement out of the 3 hospitals. The mean of the data was laying ininterval 3** **(60-80 overscanned), and after 5 months the mean was in interval 2 (20-40). Hospital B showed the second-best improvement. The mean was in interval 2 (20-40 overscanned) and after 5 months the mean of the data was in interval 1 (0-20 overscanned) (Table 6). Hospital A didn’t show improvement in the overscan percentage. The mean was in interval 2 and after 5 months it stayed in interval 2.

**Table 6:** Hospital overscan cross-tables (reaudit) Chi-square for overscan distribution in each hospital after 5 months represented as N (%) per hospital Key to overscan percentages on 5 intervals: 1=(0-20%), 2=(21-40%), 3=(41-60%), 4=(61-80%), 5=(81-100%)

		1	2	3	Total
Hospital	Hospital B	14 (56%)	11 (44%)	0 (0%)	25 (100%)
	Hospital C	5 (33.33%)	9 (60%)	1 (6.67%)	15 (100%)
	Hospital A	2 (20%)	7 (70%)	1 (10%)	10 (100%)
Total	-	21(42%)	27 (54%)	2 (4%)	50 (100%)

## Discussion

Imaging of patients presenting with symptoms of renal pathologies provides vital information for the correct diagnosis. Ultrasonography is recommended by the American Urological Association (AUA), European Association of Urology (EAU), and American College of Radiology (ACR) as the first-line imaging modality for pregnant and young patients, as there is serious concern about radiation exposure in children and young people, especially to the genitalia. However, in adults, the ACR and AUA both recommend CT as the first-line investigation for symptoms of obstructive nephrolithiasis. The sensitivity and specificity of CT scans are 95% and 98% respectively, which is proved by a recent review to be of higher values than all other imaging modalities, including MRI, ultrasound, or plain radiographs [9].

In this study, data have been collected from 88 patients, and the results of each patient have been compared with the standard criteria. A notable criteria non-compliance was detected, which indicated excess scan length as exceeding 10% of the total length of the scan, above the upper pole of the highest kidney. In our centers, 100% of patients did not meet the best practice guidelines for renal CT imaging. As a result, more imaging scans were conducted to reach an accurate diagnosis, leading to elevated exposure to harmful radiation. Additionally, slices in the axial plane were documented beyond the point where the uppermost pole of the kidney had been identified.

A thorough literature search identified only two pertinent articles in the realm of quality improvement and audit, aiming to enhance the quality of renal CT imaging modalities. The search, conducted on Pubmed up to 2022, focused on English articles under the mesh Clinical Audit, using keywords CT renal and audit. Non-relevant articles, such as those discussing indications for renal CT or optimal radiological doses, were excluded from the current review. A summary was compiled for the relevant audit articles found (Table 7).

**Table 7:** A comparison between the published studies CT-KUB: Computed tomography of kidneys, ureters, and bladder; DLP: Dose length product

	Author	Cycle	Quality standard	No. of cases	Overscan percentage	Exposure	Position	Improvements methods
1	Netke et al. [8]	Audit	The number of CT-KUB slices above the upper pole of the highest kidney, relative to scan length	100	81%	NA/-	Top of the highest kidney	Informing the radiographers that they have the option to observe renal CT images in real-time and manually halt the scanner when the top of the highest kidney is visible, along with acquiring images in the caudocranial direction
		Reaudit	The number of CT-KUB slices above the upper pole of the highest kidney, relative to scan length	50	14%	NA/-	Top of the highest kidney	No extra training was needed as the radiographers already possessed the skills and knowledge required for caudocranial scanning and renal anatomy
2	Kasi et al.^.^[9]	Single cycle audit No reaudit available	Scan extent compliance at the superior (kidneys) and inferior (pubic symphysis) borders, compliance and non-compliance (over-/under-scanning), described (superior/inferior), quantified (via IMPAX measurements), PBU40 phantom	150	4 cm to 5 cm (n = 19, ~10% DLP extra), followed by 1 cm to 2 cm (n = 18, ~4.2% DLP extra), and 3 cm to 4 cm (n = 17, ~7.4% DLP extra), inferiorly>was 8 cm to 9 cm, resulting in a ~ 38.6% extra dose (DLP) to the patient	NA/-	At the kidneys by 4 cm to 5 cm, inferiorly at the pubic symphysis by 1 cm to 2 cm	By education, trialing. and implementing anatomical landmarks

Previous reviews did not employ identical data collection parameters. Netke et al., in their study employed metrics such as the total number of additional slices, slices encompassing both kidneys, the distance from the beginning of the scan to the upper pole of the highest kidney, along with the resulting percentage of "overscan" [8]. Identification of the uppermost renal point on axial imaging was performed to calculate additional scans, involving a comparison between slices above this point and the total axial slices. In the study by Kasi et al., IMPAX measurements were integrated as part of the assessment [9].

In the second audit cycle, Netke et al. refined the protocol by enabling radiographers to monitor renal CT images in real-time, manually halting the scanner upon sighting the top of the highest kidney in addition to caudocranial direction images [8]. Conversely, Kasi et al., employing a single-looped audit, proposed protocol improvement through education, addressing current CT scan range extension in one body region, and implementing anatomical landmarks [9].

From these scans, 63% were 20-40% overscanned, and only one patient was more than 80% overscanned. Patients with renal pathologies, especially those with recurrent indications like renal calculi, face increased exposure to ionizing radiation due to frequent renal scans. This heightened risk is especially relevant in our study population, where 30% of participants were aged between 21 and 40 years, and 55.7% were aged between 41 and 60 years. Because radiation exposure from all sources can add up over a lifetime, and radiation can increase cancer risk, imaging tests that use radiation should only be done when necessary, and protocols to reduce overscanning must be implemented. Moreover, the probability of detecting incidental pathologies was raised by the process of overscanning. Most of these findings are benign, but they contribute primarily to patient anxiety and add to the workload of healthcare professionals, as there is a mutual desire to investigate these additional results [10].

This audit has encountered multiple disparities in data collection including the potential variability in overscan assessment due to different residents reviewing CT images across the 3 included hospitals. The absence of a hospital in northern Jordan limits the geographical representation. Moreover, the relatively short data collection period in each hospital may not accurately capture the expected long-term trends. Generalizability is restricted to the Jordanian context, and the exclusive focus on Jordan prevents extrapolation to other regions. Factors like image quality, which were not extensively reviewed in this audit, could influence the interpretation of final results. It is crucial to optimize CT-KUB scanning and adhere to recommended protocols, as these scans play a fundamental role in diagnosing and monitoring renal pathologies. Achieving this optimization involves targeted re-education for specific CT radiographers regarding the accepted position, penetration, and quality of CT-KUBs. Hence, it is crucial to optimize CT-KUB scanning and adhere to recommended protocols, as these scans play a fundamental role in diagnosing and monitoring renal pathologies. Achieving this optimization involves targeted re-education for specific CT radiographers regarding the accepted position, penetration, and quality of CT-KUBs.

## Conclusions

In this study, a comprehensive examination of renal CT scans for urinary symptoms exposed a concerning trend, subjecting all 88 patients to excessive radiation. Methodologically, a retrospective clinical audit spanning three Jordanian hospitals meticulously assessed CT scan overscanning. Findings revealed universal non-compliance with guidelines, urging an immediate shift in imaging practices. The study recommends recognizing specific anatomical landmarks during scans and implementing regular audits. Results showed that none of the evaluated patients met established guidelines, emphasizing the critical need for proactive measures. The discussion underscores the broader implications, highlighting a prevalence of overscanning and proposing targeted re-education for radiographers. The dynamic nature of best practices, as gleaned from the literature review, further accentuates the urgency for continuous refinement in renal CT imaging. This study not only contributes empirical evidence but serves as a pivotal call to optimize practices for enhanced diagnostic precision and unwavering patient safety in renal CT imaging.

## Figures and Tables

**Figure 2:**
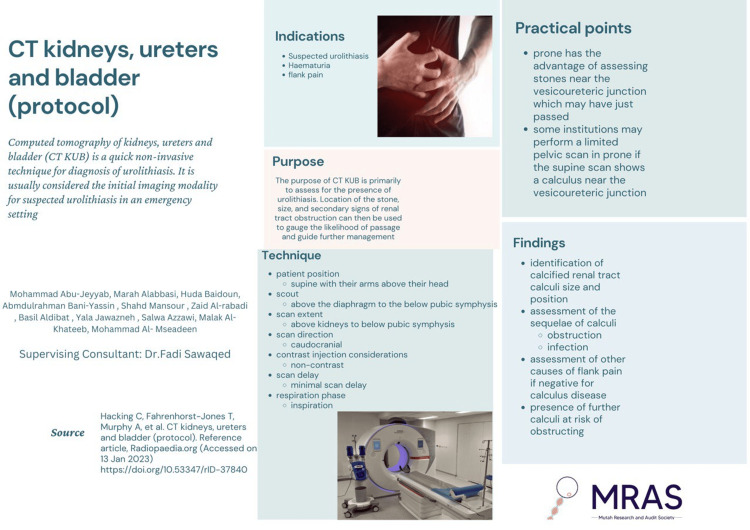
Poster used to teach the staff about the guidelines of CT-KUB CT-KUB: Computed tomography of kidneys, ureters, and bladder

**Figure 3:**
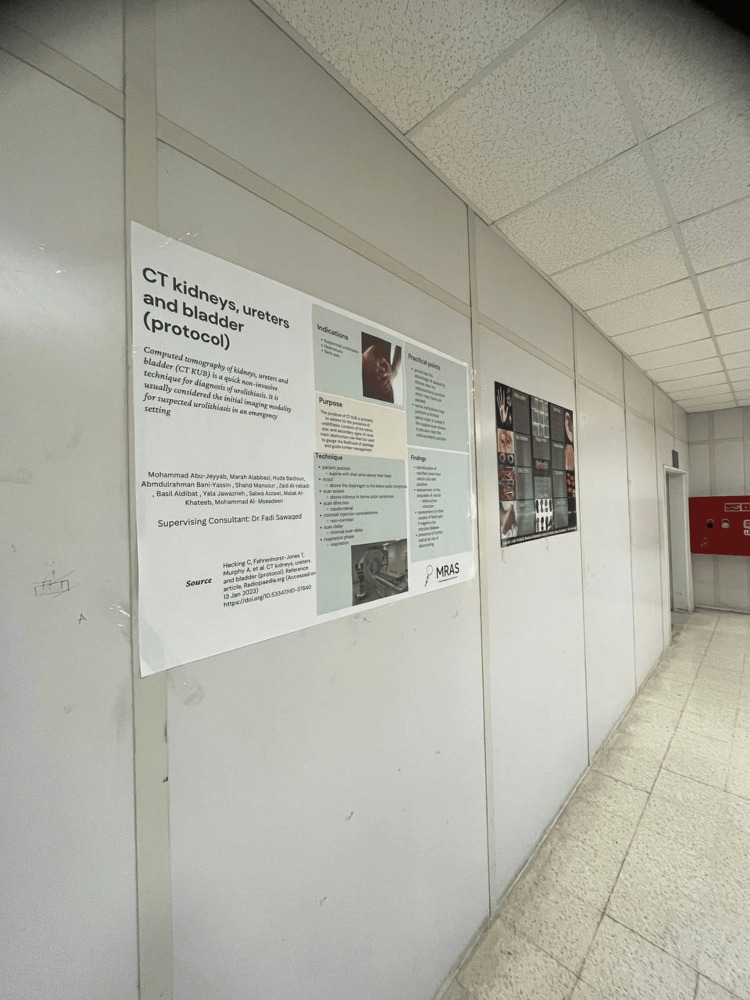
Hospital A poster view

**Figure 4:**
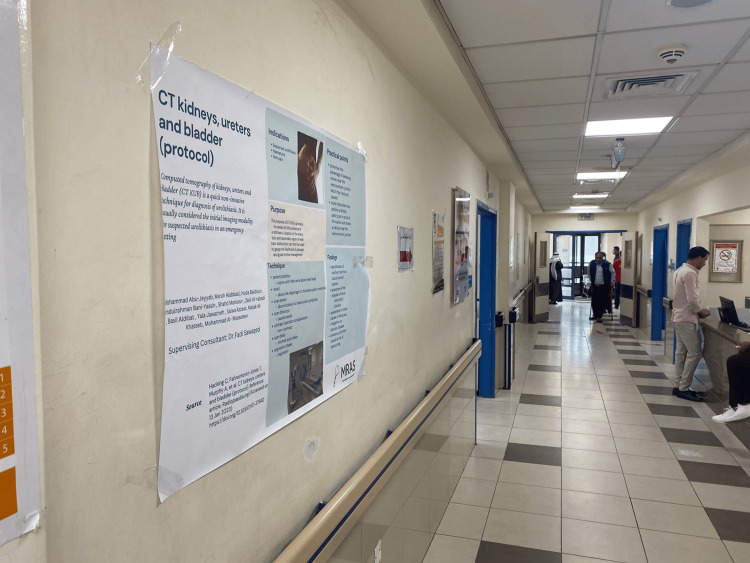
Hospital B poster view

**Figure 5:**
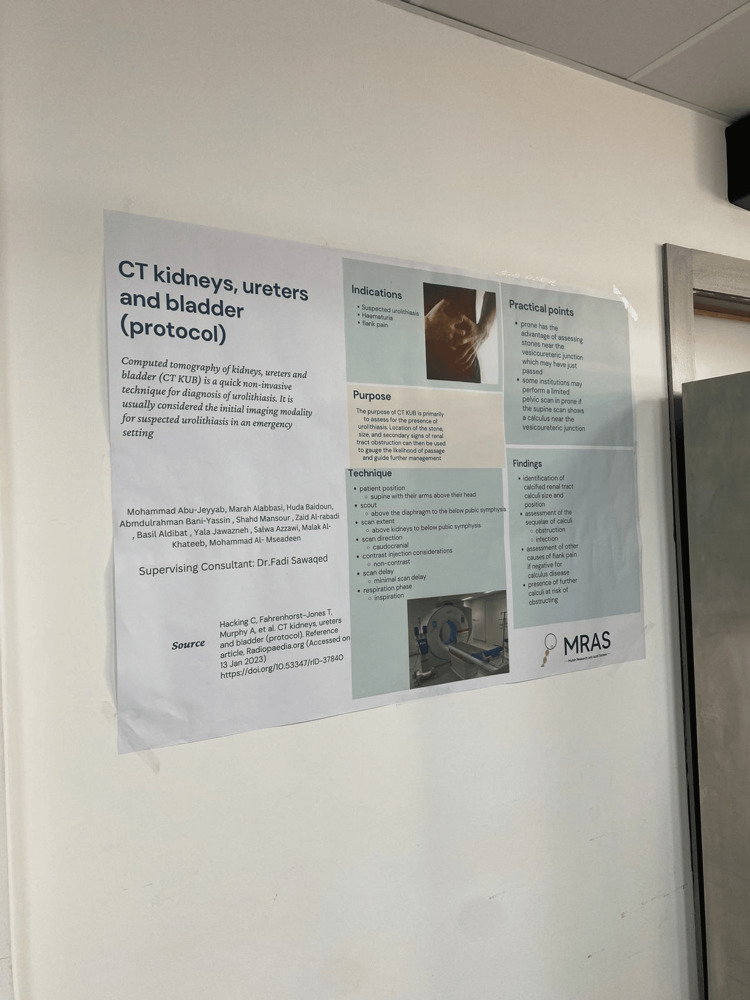
Hospital C poster view
